# IgG4-Related Hepatic Pseudotumor Masquerading as a Klatskin Tumor

**DOI:** 10.1155/2022/5765116

**Published:** 2022-06-28

**Authors:** Jennifer Yoon, Steve Hu, Daniel Phillips, Amir Fathi, Adnan Ameer

**Affiliations:** ^1^Department of Internal Medicine, University of California, Fresno, CA, USA; ^2^Department of Gastroenterology and Hepatology, University of California, Fresno, CA, USA; ^3^Department of Pathology, Community Regional Medical Center, Fresno, CA, USA; ^4^Department of Surgery, University of California, Fresno, CA, USA

## Abstract

Immunoglobulin G subclass 4 (IgG-4)-related disease (IgG4-RD) is an uncommon immune-mediated, fibro-inflammatory disease which has garnered recognition as a systemic condition. One manifestation of the disease in the hepatobiliary system is the development of hepatic inflammatory pseudotumors. These benign tumors are often misdiagnosed as malignant tumors and undergo unnecessary hepatic resections. We present a case of IgG4-related hepatic inflammatory pseudotumor (IPT) mimicking a Klatskin tumor. A high degree of clinical suspicion and extensive workup is imperative in reaching the correct diagnosis. IgG4-related inflammatory pseudotumor is a rare entity, but an important consideration in evaluating hepatic tumors.

## 1. Introduction

Inflammatory pseudotumors are benign, mass-forming lesions that consist of inflammatory cells, fibrous tissue, and myofibroblasts and are often mistaken for malignant tumors [1, 2]. They are commonly misconstrued as pancreatic cancer, Klatskin tumors, and lymphomas [3]. Hepatic IPTs can be due to viral or bacterial infections, congenital disease, gallstones, chronic biliary inflammation, and IgG4- related disease (IgG4-RD) [4]. IgG4-related hepatic IPTs are uncommon; differentiation from malignant tumors is crucial for management and avoiding unnecessary resection. Here, we describe a case of an IgG4-related hepatic IPT mimicking a Klatskin tumor.

## 2. Case Description

A 59-year-old incarcerated male with human immunodeficiency virus on highly active antiretroviral therapy presented with abdominal pain and jaundice. His liver panel was notably abnormal, with alkaline phosphatase 344 U/L (25–100 U/L), ALT 61 U/L (10–40 U/L), AST 44 U/L (8–40 U/L), total bilirubin 11.3 mg/dL (0.3–1.2 mg/dL), and direct bilirubin 7.2 mg/dL (0.0–0.4 mg/dL). An initial computed tomography (CT) scan revealed an amorphous-appearing pancreatic head mass with dilated intra- and extrahepatic bile ducts. A serum IgG4 level measured 354 mg/dL (4–86 mg/dL), and cancer antigen (CA) 19–9 was 97.9 U/ml (0–35.0 U/mL). Endoscopic ultrasound (EUS)-guided fine needle biopsy of the pancreatic head mass was performed. Pathology was consistent with type I autoimmune pancreatitis by immunohistochemistry, and no malignant cells were identified. Due to significant biliary obstruction, he underwent an endoscopic retrograde cholangiopancreatography (ERCP) with stenting of the distal inflammatory biliary stricture and initiated prednisone therapy with an extended taper, resulting in subsequent normalization of CT findings and liver labs.

**Figure 1:**
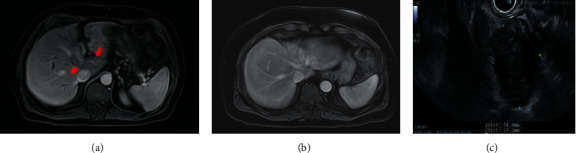
(a) Magnetic resonance imaging (MRI) of the abdomen demonstrating hepatic IPT prior to treatment with steroid taper. (b) MRI of the abdomen after treatment showing complete resolution of hepatic IPT. (c) Endoscopic ultrasound demonstrating a periportal mass.

**Figure 2:**
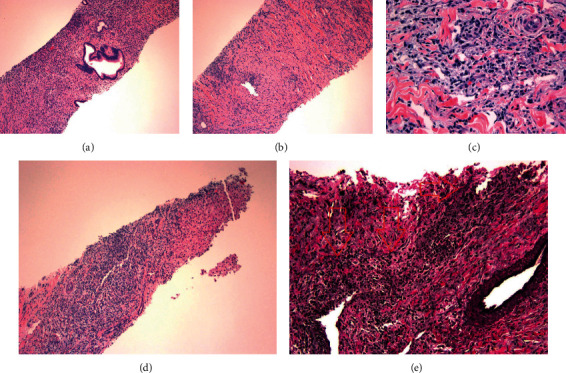
(a–d) Hepatocellular parenchyma with a mixed predominately mononuclear inflammatory infiltrate and reactive fibrosis, with residual bile duct elements present. (c) 400x total magnification; infiltrate is mainly comprised of lymphocytes and plasma cells. (d) The interface of the inflammatory process with background liver tissue. (e) Arrows highlight the residual elastic lamina.

Two years later, he again presented with similar symptoms. His liver enzymes were again elevated in a cholestatic pattern with a total bilirubin of 4.0 mg/dL, and his serum IgG4 was 458.9 mg/dL. The CT scan showed a mass-like mural thickening at the hepatic hilum concerning for a Klatskin tumor (Figure 1). EUS confirmed an irregular hypoechoic mass in the porta hepatis measuring 4.1 × 2.7 cm (Figure 1). Histopathology showed dense lymphoplasmacytic infiltrating and a fibrosing process with residual bland pancreatic ducts and lobules. Immunohistochemistry demonstrated IgG4 plasma cell positivity (>10 per high-power field (HPF)). A subsequent CT-guided biopsy of the hilar mass also confirmed IgG4-positive plasma cells and obliterative phlebitis, consistent with IgG4 -associated hepatic IPT (Figure 2). He was again treated with a tapered course of prednisone with repeat imaging showing complete resolution of the pseudotumor.

## 3. Discussion

IgG4-RD is a chronic, immune-mediated, fibro-inflammatory disease that can involve multiple organ systems and often mimics malignancy, infection, or infiltrative disease. It is predominant in middle-aged to elderly men and commonly affects the pancreas, salivary glands, orbit, kidneys, and retroperitoneum [5, 6]. Diagnosis is based on a comprehensive workup of serology, imaging, histopathology, and response to steroids. While a histologic diagnosis was previously required, a clinical diagnosis can now be made using the 2019 American College of Rheumatology (ACR)/European League Against Rheumatism (EULAR) Executive Committee classification criteria [7]. Patients who meet the entry criteria in the absence of the exclusion criteria with a score of ≥20 are classified as having IgG4-RD, our patient scored a total of 38 points [7]. Histologically characteristic findings of IgG4-RD include a lymphoplasmacytic infiltrate, heavy IgG4-positive plasma cell infiltration, storiform fibrosis, and obliterative phlebitis [4, 7]. The most common manifestation of IgG4-RD in the hepatobiliary system is cholangitis; rarely, hepatic IPTs can develop as a late-stage manifestation of the disease [8]. Due to the mass-forming nature of these lesions and nonspecific findings on imaging, these benign tumors are often mistaken for malignant tumors.

Traditionally, the first-line treatment has been systemic steroids, although some cases report spontaneous resolution [9,10]. Treatment is indicated for symptomatic patients or asymptomatic patients with progressive disease. Early recognition and prompt treatment are preferred due to concern for end-organ damage. Weight-based dosing of steroids, typically prednisone, is used to induce remission usually at a dose of 0.6 mg/kg/day for 2–4 weeks and gradually tapered off over the course of months depending on response to therapy [8, 11]. Use of systemic steroids may be limited due to side effects including poor glycemic control, infection, and weight gain [4]. Immunosuppressive agents such as rituximab, azathioprine, or mycophenolate mofetil may be used as adjunct agents for patient's intolerant systemic steroids due to significant side effects, nonresponse, or relapsing disease [8, 11]. More recently, rituximab has emerged as the preferred treatment due to a better side-effect profile with comparable efficacy to systemic steroids [12, 13].

Misclassification of hepatic IPTs for malignant tumors has resulted in unnecessary hepatic resections. IgG4-related hepatic IPTs are predominant in middle-aged to elderly males, a demographic which is more likely to lead physicians to suspect underlying malignancy. Our literature review has shown a total of 46 (36 males and 10 females) reported cases of hepatic IPTs, excluding this case (Table 1). Of the reported cases, initial diagnostic impressions were widely variable. Approximately 67% of the reported cases perceived the pseudotumor to be an underlying malignancy resulting in resections in 81% of cases. Of the 46 reported cases, 16 cases underwent a biopsy. Despite a negative biopsy result, still 12.5% of these cases underwent unnecessary liver resections. Of the patients who underwent biopsy with confirmed histopathologic diagnosis, 87.5% were spared surgical resections.

IgG4-related hepatic IPTs are rare and pose a diagnostic challenge given their resemblance to malignant tumors. A high degree of clinical suspicion and thorough workup is imperative in preventing patient harm from unnecessary resections. An accurate diagnosis can be established through tissue sampling with biopsy, and subsequently, hepatic resections can be circumvented. Our case details the definitive diagnosis and treatment of IgG4-associated hepatic IPT with successful avoidance of surgery. In addition, as IgG4-RD is a chronic disease, the development of the hepatic IPT may have been avoidable if the patient had been diagnosed with IgG4-RD at his first presentation and was sustained on maintenance therapy.

## Figures and Tables

**Table 1:** The literature review of reported IgG4-related hepatic IPTs to date.

Author	Year	Age	Sex	Tumor number/location	Biopsy	Biopsy method	Resection	Initial diagnostic impression	Serum IgG4
Adachi K [14]	2017	60	M	1/Segment 3	N		Y	Hepatic cancer	Elevated
Adachi Y [15]	2020	80s	F	1	Y	Unspecified	N	Hepatic IPT	Elevated
Ahn KS [9]	2012	58	M	1			Y	Recurrence of Klatskin tumor	
		60	M	1			Y	Malignancy	
		76	M	2			N	Hepatic IPT	
		52	M	1			N	Hepatic IPT	
Buchter M [16]	2018	58	M		Y	Unspecified	N	Hodgkin's lymphoma	2528
Chia JS [17]	2020	53	M		N		Y		
Chung JW [18]	2019	67	M	1	Y	Percutaneous	Y	Abscess, cancer	2215
Fujisaki H [19]	2016	75	F	1/Segment 7	N		Y	Hepatic IPT	Serum normal, bx elevated
Hamano A [6]	2020	71	M	1/Segment 3	Y	Percutaneous	N	Hepatic cholangiocarcinoma	180
Hastir D [20]	2013	50	F	1/Segment 5	N		Y	Hepatocellular carcinoma	
Horiguchi S [21]	2012	76	M	1/Segment 2	Y	Laparoscopic	N		819
Itazaki Y [2]	2021	75	F	1			Y	Cholangiocarcinoma	
Kanno a [22]	2005	48	M		N		Y	Liver metastasis from cholangiocarcinoma	2150
Kataoka K [23]	2017	79	M		Y	Unspecified	N		Elevated
Kim F [24]	2011	58	M	1/Segment 4	Y	Percutaneous	N		1470
Lee YS [25]	2013	59	M	1/Segment 5	Y	Percutaneous	N	Inoperable gallbladder cancer	75
Legkiy O [26]	2019	60	M	2/Segment 2 & 6	Y	Percutaneous	Y		19.1
Matsuo Y [27]	2014	74	M	1/Segment 8	N		Y	Liver metastasis from GIST	
Miyajima S [28]	2017	50	F	1	Y	Unspecified	N	Cholangiocarcinoma	241
Mulki R [29]	2015	50	M	2	N		Y	Abscess with impending rupture	>182
Naitoh I [30]	2009	77	M	1/Segment 3	N		Y	Intrahepatic cholangitis	231
Patel H [10]	2018	48	M	1	Y	Unspecified	N		2228
Shibata M [4]	2016	72	M	1/Segment 7	Y	Percutaneous	N		137
Uchida K [31]	2007	54	M	1/Segment 4	Y	Unspecified	N	Pancreatic cancer with liver metastasis	213
Vadi S [32]	2018	54	M	1/Gallbladder fossa	Y	Unspecified	N		356
Wang M [33]	2020	78	M	1/Segment 4	N		Y	Cholangiocarcinoma	
Yang L [34]	2015	60	M	1/Segment 3	Y	Unspecified	N		1590
Yueh HZ [35]	2021	47	M	Multiple	Y	Percutaneous	N	Metastatic cancer	1786
Zen Y(16 cases) [36]	1990–2005	Avg 67 (56–82)	11M 5F		N		Y	(All resected)	Hilar cholangiocarcinoma (8), intrahepatic cholangiocarcinoma (5), malignant hepatic tumor, undetermined origin (2), metastatic carcinoma (1)
